# Retrospective Analysis of RSV Infection in Pediatric Patients: Epidemiology, Comorbidities, Treatment, and Costs in Dubai (2014-2023)

**DOI:** 10.36469/001c.123889

**Published:** 2024-11-05

**Authors:** Jean Joury, Nawal Al Kaabi, Sara Al Dallal, Bassam Mahboub, Mostafa Zayed, Mohamed Abdelaziz, Jennifer Onwumeh-Okwundu, Mark A. Fletcher, Subramanyam Kumaresan, Badarinath C. Ramachandrachar, Mohamed Farghaly

**Affiliations:** 1 Pfizer Gulf FZ LLC, Dubai, UAE; 2 College of Medicine and Health Science, Khalifa University, Abu Dhabi, UAE; 3 Sheikh Khalifa Medical City, Abu Dhabi Health Services Company (SEHA), Abu Dhabi, UAE; 4 Dubai Health Authority, Dubai, UAE; 5 Pfizer Inc., Memphis, Tennessee, USA; 6 Pfizer Inc., Paris, France; 7 EMEA Consulting Services, IQVIA, Bengaluru, India; 8 Real-World Evidence, IQVIA, Dubai, UUAE; 9 Health Economics & Insurance Policies Department Dubai Health Authority, Dubai, UAE

**Keywords:** respiratory syncytial virus, infants, lower respiratory tract infection, hospitalization, palivizumab, immunoprophylaxis, e-claims database

## Abstract

**Background:** Infections attributable to respiratory syncytial virus (RSV) are a major cause of hospitalization among young children worldwide. Despite substantial clinical and economic burden, real-world data associated with RSV infections in the United Arab Emirates (UAE) are limited. **Objectives:** This study aimed to assess among children (<18 years) diagnosed with RSV the epidemiology, seasonality, comorbidities, treatment patterns, length of hospital stay, healthcare resource utilization (HCRU), and costs associated with pediatric infection in Dubai, UAE. **Methods:** This 10-year retrospective cohort study (Jan. 1, 2014–Sept. 30, 2023) utilized Dubai Real-World Database, a private insurance claims database. Patients aged <18 years with a first-episode diagnosis claim (primary or secondary, or a hospital admission) for RSV any time during the index period (Jan. 1, 2014–June 30, 2023) were included. Outcomes were analyzed during a 3-month follow-up. Patients were stratified into 3 cohorts: Cohort 1 (<2 years), Cohort 2 (2 to <6 years), and Cohort 3 (6 to <18 years). **Results:** Of 28 011 patients identified, 25 729 were aged <18 years with RSV infection. An increasing trend in reported cases was observed from 2014 to 2022, with an average annual increase of 55%. Half of study patients (49.3%) belonged to Cohort 1, with a mean age of 0.6 years, while less than 2% had known risk factors and 22% of the patients in cohort 1 were hospitalized. In Cohort 1, 32.0% had upper respiratory tract infections, 39.4% had lower respiratory tract infections, and 44.4% of patients had an “other respiratory disease.” The average length of hospitalization was about 4 days across all cohorts. The total hospitalization cost was highest in patients <2 years, amounting to US 9 798 174(median,US2241.30). **Conclusion:** Among the RSV patients, 49.3% were <2 years of age and few had recognized risk factors. Among patients <2 years, 22% were hospitalized, with an average hospital stay of 4 days; the cost of hospitalization totaled US $9 798 174. These findings can inform healthcare stakeholders about future policy measures and the need for effective preventive strategies.

## INTRODUCTION

Respiratory syncytial virus (RSV) is a dominant cause of acute lower respiratory tract infections (LRTIs) and burden of respiratory tract infections (RTIs) among young children worldwide, as substantiated by hospital admissions and mortality. An RSV infection occurs at least once in the first 2 years of life among 90% of children, with clinical signs and symptoms ranging from mild coryzal illness and upper respiratory tract infections (URTIs), and severe RTIs such as bronchiolitis and pneumonia. These conditions can be compounded by viral-induced wheeze and exacerbation of asthma.^1–3^ In 2019, it was assessed that among children aged up to 60 months, there were 33 million cases of RSV-associated LRTIs (RSV-LRTIs), globally, resulting in an estimated 3.6 million hospital admissions and 26 300 in-hospital deaths. For infants (≤6 months), the estimate was 6.6 million RSV-LRTI episodes, 1.4 million hospital admissions, and 13 300 in-hospital fatalities.^4,5^ Another study, from the Middle East and North African (MENA) region, reported a 24.4% overall annual prevalence of RSV infection during 2001-2019 across all age groups.^6^ In the United Arab Emirates (UAE), RSV infection was documented among 18.79% of infants under 2 years diagnosed with acute RTI.^7^

The risk of hospitalization and mortality attributable to RSV is higher among children with risk factors such as age (<6 months during the RSV season), male sex, prematurity (gestational age <37 weeks or low birth weight), or underlying medical conditions at birth (eg, trisomy 21 or congenital anomalies, including bronchopulmonary dysplasia or congenital heart disease).^8–13^ RSV infection incidence can vary by season; for instance, in the UAE, the largest number of infections each year were reported in winter, with a peak during October.^7,14^

Currently, the mainstay of RSV treatment is supportive, including respiratory and nutritional care.^2,5^ In the last few decades, neonates and infants in high-risk categories have received passive immunization with palivizumab, a monoclonal antibody, to reduce the likelihood of RSV infection and RSV-related sequelae.^9^ Given the high RSV burden, as well as the seasonality of the disease, it is imperative that novel strategies are explored to combat the disease. In this regard, the European Medicines Agency’s approval of Abrysvo™ for active maternal immunization to provide passive protection against RSV-LRTI among neonates and young infants (ie, ≤6 months) is a key milestone.^15^ The Dubai Health Authority also recommends immunoprophylaxis with palivizumab among high-risk infants to reduce RSV-related hospitalizations. Recently, nirsevimab, a longer-duration monoclonal antibody therapy, has been developed as a preventive strategy against RSV for all infants.^9,16^

Apart from having a substantial disease burden, RSV infection also imposes a heavy economic burden. A retrospective analysis of RSV-associated hospitalization data among children under 5 years of age from a national French hospital database of secondary and tertiary care from the public and private sectors indicated that the cost of RSV-related hospitalization ranged from €93.2 million in 2010-2011 to €124.1 million in 2017-2018, with infants under 1 year old accounting for 80% of the total cost burden.^17^ In an observational retrospective study conducted using anonymized administrative public hospital discharge data from Spain, the mean annual direct healthcare cost for RSV infection was reported at €87.1 million among children aged under 5 years, with hospitalization being a major driver of these costs.^18^

Despite this significant clinical and economic burden, real-world data in the UAE on disease burden and healthcare costs associated with RSV-attributable infections are limited. This study, using an e-claims database, aims to evaluate the disease burden (epidemiology, comorbidity burden, treatment patterns, hospitalization) and healthcare costs (healthcare resource utilization [HCRU] and associated costs) among RSV-infected patients in Dubai, UAE.

## METHODS

### Study Design

**Figure 1.**
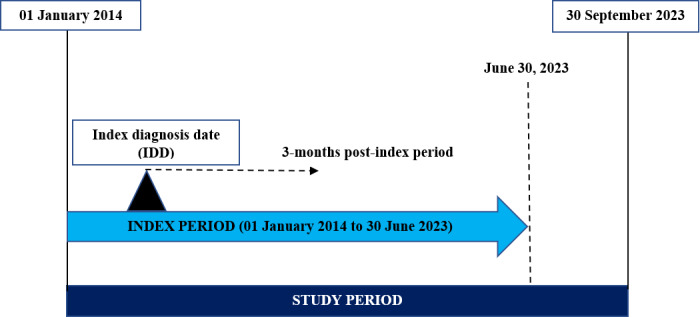
Overview of Study Design Study design where the study period and index period have been specified. Post-index (follow-up) period varied for each patient based on the index diagnosis date.

The current study is a retrospective observational cohort study conducted using Dubai Real-World Database (DRWD) e-claims data from January 2014 to September 2023. The date of the first diagnosis of RSV infection was termed the index diagnosis date. The index period for the analysis was from January 2014 to June 2023. A follow-up period from the index diagnosis date was regarded as the post-index period (**Figure 1**).

### Data Source

The DRWD e-claims database is the largest patient-level database encompassing insurance-related claims covered by private insurance in the Emirate of Dubai. The public healthcare system is primarily utilized by Emirate nationals, whereas expatriates, who constitute about 89% of the total population, are covered by private insurance. This database includes approximately 100% of the population covered by Dubai private insurance and primarily reflects the diverse expatriate population. The DRWD e-claims database includes comprehensive information regarding patient demographics, diagnoses, medical procedures (including medical and surgical, as well as diagnostic), prescriptions, additional related services, treatments, and consultations.

### Study Population

The *International Classification of Diseases, Tenth Revision, Clinical Modification* (ICD-10-CM) codes were used to identify patients with RSV infection. The codes selected for RSV diagnosis (B97.4, J12.1, J20.5, and J21.0) are elaborated in **Table S1**. Patients with at least 1 RSV diagnosis claim during the index period were included in the analysis. Patients with coronavirus infection (ICD-10 codes: U07.1, U07.2, B34.2), those ≥18 years of age, and those with no age details available were excluded.

The included pediatric patients were further stratified into 3 cohorts based on age:

- Cohort 1: <2 years
- Cohort 2: 2 to <6 years
- Cohort 3: 6 to <18 years

### Ethical Considerations

The study objective and methodology were reviewed in collaboration with Dubai Health Authority, and the required approvals were obtained.

Patient-level data in the database are anonymized to comply with Dubai/UAE specific data protection regulations. Use of this database for health services research is fully compliant with Dubai/UAE specific laws, and accordingly, Institutional Review Board/ethical approval was not required because all patient-level data in the database are anonymized. The study conformed with the Helsinki Declaration of 1964, as revised in 2013, concerning human and animal rights. Informed consent was not required as this analysis used de-identified claims data.

### Baseline Patient Characteristics and Outcomes

Patient demographics and insurance plans: Patient age across the 3 cohorts and insurance plans were determined at the index date (date of first diagnosis of RSV); the patient’s sex and nationality were determined based on the latest available information from the e-claims data.

**RSV claims:** The total number of patients with RSV claims (as per ICD-10 codes) reported during the period from January 1, 2014, to December 31, 2022, was considered for reporting data for the proportion of patients with RSV year-over-year.

**Three-month post-index period assessments**: During the 3-month post-index period, the following study variables were assessed across all 3 cohorts: comorbidities of interest (including risk factors), where ICD-10 codes were used to identify the comorbidities of interest and risk factors; frequently prescribed treatment patterns; and duration of hospital stay. Among infants, the distribution of the proportion of patients hospitalized by age in months was also analyzed.

**RSV seasonal variability**: RSV seasonal variability in Dubai was assessed from January 2014 to December 2022. Since RSV cases tend to increase during the winter, in the last year of the study period, the seasonality of RSV was evaluated up to December 31, 2022, rather than the end of the study period on September 30, 2023, to encompass the peak months of RSV incidence.

**HCRU and associated costs:** HCRU and associated costs (all-cause and disease-specific) were evaluated for all cohorts. The all-cause cost incurred included the healthcare costs incurred for all the claims (visit/medication/consultation/consumables) that patients with an ICD-10 code of RSV encountered during the follow-up period; the disease-specific cost included healthcare costs incurred for claims (visit, medication, consultation, and consumables) specific to RSV, with or without other diagnosis code within the same claim, during the follow-up period. Patient records were assessed by visit type (inpatient visits, emergency department (ED) visits, and outpatient visits) and activity type (medications, procedures [medical, surgical, and diagnostic services], consumables [medical and surgical supplies], services [administrative and consultations], and diagnosis-related group [DRG]). Gross cost and net cost were obtained from HCRU data. The percentage of disease burden was also estimated.

**Intensive care unit costs:** The cost of intensive care unit (ICU) admission during the 3-month post-index period was evaluated and reported in terms of gross cost. ICU costs included costs for any admission in the ICU, neonatal intensive care unit (NICU), or pediatric intensive care unit (PICU).

### Statistical Analysis

Descriptive statistics were used to analyze and report the study variables. Categorical variables, including sociodemographics, comorbidities (including risk factors), and treatment patterns, were summarized by number and proportion summarized as a percentage. The ages of patients (overall and per age category) were reported as means, standard deviation (SD), medians, minimums, and maximums. Continuous variables, including HCRU and cost, ICU cost, and the length of stay, were summarized by providing the means, SD, medians, 25th and 75th percentiles, interquartile ranges, minimums, and maximums, as appropriate, based on the number of observations.

Data for RSV seasonal variability and the proportion of patients with RSV year-over-year were summarized.

In calculating HCRU and costs, and assessing the comorbidities of interest (including risk factors) and treatment patterns, a patient might have been present at more than 1 encounter or activity type. Hence, the patient counts were not mutually exclusive.

All analyses were performed by IQVIA’s Real-World Data team using Microsoft SQL Server 2014 and SAS® version 9.4.

## RESULTS

### Demographic Characteristics of Patients With RSV Infection

Of the total of 28 011 patients with RSV, the study identified that 25 729 (91.8%) were under 18 years of age. Data on sex and nationality for privately insured patients in the e-claims database were available for 54% and 18%, respectively.

The proportion of patients in each cohort was: 49.3% in Cohort 1 (<2 years of age, n = 12 683); 41.1% in Cohort 2 (2 to <6 years of age, n = 10 573); and 9.6% in Cohort 3 (6 to <18 years, n = 2473). The mean ages were 1 year for Cohort 1, 3 years for Cohort 2, and 8 years for Cohort 3. Across all 3 cohorts, the proportion of female patients ranged from 45% to 49%. The study patients were primarily of the following nationalities: Indian (41%-60%), Filipino (9%-13%), Pakistani (6%-7%), Egyptian (4%-6%), and Jordanian (2%-5%) (**Table 1**).

**Table 1.** Sociodemographic Characteristics of Patients With RSV

**Parameter**	**Cohort 1**	**Cohort 2**	**Cohort 3**
No. (%) of patients	12,683 (49.3)	10,573 (41.1)	2473 (9.6)
Age (years)			
Mean (SD)	0.6 (0.5)	3.0 (1.0)	7.9 (2.3)
Median (range)	1 (0-1)	3 (2-5)	7 (6-17)
Sex, n (%)			
Data available	5770 (45.5)	5566 (52.6)	1574 (63.6)
Female	2739 (47.5)	2746 (49.3)	705 (44.8)
Male	3031 (52.5)	2820 (50.7)	869 (55.2)
Unknown/NA	6913	5007	899
Insurance type, n (%)			
Data available	1836 (14.5)	2145 (20.3)	479 (19.4)
Basic	441 (24.0)	407 (19.0)	95 (19.8)
Premium	1395 (76.0)	1738 (81.0)	384 (80.2)
Unknown/NA	10847	8428	1994
Nationality, n (%)			
Data available	1836 (14.5)	2144 (20.3)	479 (19.4)
India	758 (41.3)	1128 (52.6)	289 (60.3)
Philippines	226 (12.3)	279 (13.0)	45 (9.4)
Pakistan	128 (7.0)	135 (6.3)	33 (6.9)
Egypt	102 (5.6)	78 (3.6)	21 (4.4)
Jordan	84 (4.6)	48 (2.2)	12 (2.5)
UAE	79 (4.3)	55 (2.6)	14 (2.9)
Other	459 (25.0)	421 (19.6)	65 (13.6)
Unknown/NA	10847	8429	1994

Cohort 1, <2 years of age, n = 12 683; Cohort 2, 2 to <6 years of age, n = 10 573; Cohort 3, 6 to <18 years, n = 2473.

Abbreviations: NA, data not available; RSV, respiratory syncytial virus, SD, standard deviation; UAE, United Arab Emirates.

### Proportion of Patients With RSV: Year-Over-Year Trend in Distribution of Cases

Analysis of the year-over-year distribution of RSV cases showed an increasing trend with an increase of 55% observed between 2014 to 2022. However, a slight decline was noted in 2020, followed by a sharp upsurge.

### Clinical Diagnosis and Risk Factor Analysis in Patients With RSV Infection

During the 3-month post-index period, information on RSV-associated clinical diagnoses was available for 78% of the study patients (n = 20 071).

In Cohort 1, 32.0% had URTIs (n = 4052), 39.4% had LRTIs (n = 4992), and 44.4% had “other respiratory disease” (n = 5635). In Cohort 2, 38.2% had URTIs (n = 4042), 50.2% had LRTIs (n = 5309), and 45.9% had “other respiratory disease” (n = 4855). In Cohort 3, 50.1% had URTIs (n = 1240), 62.2% had LRTIs (n = 1540), and 55.0% (n = 1361) had “other respiratory disease” (n = 1361). The primary risk factors in Cohort 1 included congenital disorders (1.21%, n = 154), immunodeficiency disorders (1.14%, n = 145), neonatal complications (0.28%, n = 36), low birth weight (0.17%, n = 21), and complications of pregnancy (0.01%, n = 1) (**Table 2**).

**Table 2.** Comorbidities of Interest and Risk Factors and Treatment Patterns Across Subcohorts

**Comorbidity**	**Cohort 1 (N = 12 683)**	**Cohort 2 (N = 10 573)**	**Cohort 3 (N = 2473)**
No. (%) of patients with comorbidity data available	11,618 (91.6)	9815 (92.8)	2302 (93.1)
Comorbidities, n (%)			
Other respiratory disorders	5635 (44.43)	4855 (45.92)	1361 (59.1)
LRTI	4992 (39.36)	5309 (50.21)	1540 (66.9)
URTI	4052 (31.95)	4042 (38.23)	1240 (53.9)
Asthma	1144 (9.02)	994 (9.40)	229 (9.9)
Electrolyte imbalance	940 (7.41)	830 (7.85)	97 (4.2)
Other infections	321 (2.53)	266 (2.52)	55 (2.39)
Risk factors for RSV infection, n (%)			
Congenital disorders	154 (1.21)	101 (0.96)	12 (0.5)
Immunodeficiency disorders	145 (1.14)	175 (1.66)	58 (2.35)
Neonatal complications	36 (0.28)		
Low birth weight	21 (0.17)	-	-
Complications of pregnancy	1 (0.01)	-	-
Treatment pattern, n (%)			
Acetaminophen (paracetamol)	5309 (42)	4948 (47)	1263 (63.5)
Sodium chloride	5101 (40)	2871 (27)	381 (19.2)
Salbutamol (as sulfate)	4310 (34)	2912 (28)	362 (18.0)
Sea water	3090 (24)	1696 (16)	327 (16.0)
Budesonide	3007 (24)	2428 (23)	368 (19.0)
Ibuprofen	1647 (13)	2327 (22)	429 (21.6)
Xylometazoline HCl	662 (5)	1931 (18)	691 (34.8)
Palivizumab	82 (1)		-

Cohort 1, <2 years of age, n = 12 683; Cohort 2, 2 to <6 years of age, n = 10 573; Cohort 3, 6 to <18 years, n = 2473.

Note: Patient counts are not mutually exclusive.

Abbreviations: LRTI, lower respiratory tract infection; RSV, respiratory syncytial virus; URTI, upper respiratory tract infection.

### Treatment Patterns in Patients With RSV Infection

Of the 25 729 eligible patients, treatment data were available for 78.1% (n = 20 104).

The most prescribed medication across all 3 cohorts was acetaminophen (paracetamol) (42% in Cohort 1, 47% in Cohort 2, 51% in Cohort 3). In Cohorts 1 and 2, other frequently prescribed medications included sodium chloride (40% and 27%, respectively), salbutamol sulfate (34% and 28%, respectively), and budesonide (24% and 23%, respectively). Sodium chloride was used for intravenous infusion or for irrigation of the respiratory tract. In Cohort 1, the prescription of palivizumab was low (1.0%). In Cohort 3, other commonly prescribed medications included xylometazoline hydrochloride (28%), ibuprofen (17%), and chlorpheniramine/acetaminophen (paracetamol)/pseudoephedrine (15%) (**Table 2**).

### Length of Hospitalization in Patients With RSV Infection

During the 3-month post-index period, 2826 patients (22.3%) in Cohort 1, 1923 patients (18.9%) in Cohort 2, and 149 patients (6.0%) in Cohort 3 were hospitalized due to RSV infection. The average length of hospital stay was about 4 days for each cohort. The median length (range) of hospital stay was 4.0 (1.0-95.0) days for Cohort 1, 3.0 (1.0-130.0) days for Cohort 2, and 3.0 (1.0-38.0) days for Cohort 3 (**Table S3**).

Distribution of RSV-associated hospitalization in infants by age in months: Among Cohort 1 infants, RSV-associated hospitalization was highest in the neonatal and young infant period (≤1 month of age [20.1%, n = 581], 1-2 months of age [18.5%, n = 522], and 2-3 months of age [8.9%, n = 252], with an average length of hospital stay of 4.8 days, 4.2 days, and 4.0 days, respectively (**Figure S1; Table S4**).

### Seasonal Variability of RSV

**Figure 2.**
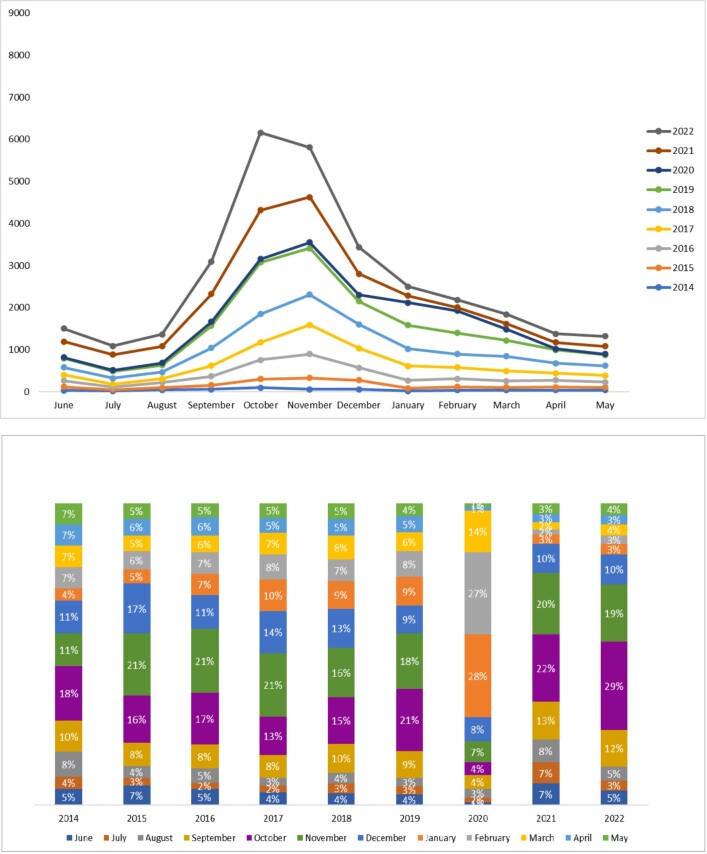
Respiratory Syncytial Virus Patient Population Distribution *Upper panel:* Patient distribution year-over-year, January 1, 2014–December 31, 2022; *lower panel:* patient population distribution month-by-month, January 1, 2014–December 31, 2022. Abbreviation: RSV, respiratory syncytial virus; SD, standard deviation.

On average, RSV cases annually increased by 55% between 2014 and 2022. The RSV season was observed from September to December, with cases surging each year starting in September (8.0%-13.0%) and peaking in October (13.0%-29.0%), November (11.0%-21.0%), and December (9.0%-17.0%). However, in 2020 a change in the trend was observed (**Figure 2**).

### HCRU and Associated Costs

**Overall HCRU and associated costs:** During the 3-month post-index period, the total costs incurred regarding overall HCRU were as follows: Cohort 1: US $16 210 311.90 (median, US $524.30); Cohort 2: US $11 618 115.90 (median, US $432.90); and Cohort 3: US $1 763 128.90 (median, US $317.00) (**Table S5**). Disease-specific costs were highest for patients in Cohort 1 (US $10 030 244.10 [median, US $192.20]), followed by those in Cohort 2 (US $6 958 900.20 [median, US $192.00]) and Cohort 3 (US $901 622.80 [median, US $171.70]).

HCRU and associated costs based on visit type: For all-cause HCRU, total claims in Cohort 1 were highest for outpatient visits (68 345 claims), followed by ED visits and inpatient visits (3699 claims each). All-cause HCRU cost in Cohort 1 was highest for inpatient visits (US $9 798 173.90 [median, US $2241.30]), followed by outpatient visits (US $5 685 795.80 [median, US $339.30]) and ED visits (US $726 342.20 [median, US $229.50]).

For disease-specific HCRU, total claims in Cohort 1 were highest for outpatient visits (19 854 claims), followed by inpatient visits (2900 claims) and ED visits (1279 claims). Inpatient visits accounted for the largest portion of the total disease-specific HCRU cost in Cohort 1 (US $7 858 582.70 [median, US $2241.20]), followed by outpatient visits (US $1 872 400.40 [median, US $140.50]) and ED visits (US $299 260.90 [median, US $250.10]) (**Table 3**). HCRU and costs for Cohorts 2 and 3 are summarized in **Table 3**.

**Table 3.** HCRU and Cost in Patients With RSV During 3-Month Post-index Period Based on Visit Type

	**All-cause Claims**			**All-cause Cost (USD)**			**Disease-Specific Claims**			**Disease-Specific Costs (USD)**		
	**Cohort 1**	**Cohort 2**	**Cohort 3**	**Cohort 1**	**Cohort 2**	**Cohort 3**	**Cohort 1**	**Cohort 2**	**Cohort 3**	**Cohort 1**	**Cohort 2**	**Cohort 3**
**Inpatient HCRU and cost**												
N (%)	3347 (26.4)	2293 (21.7)	198 (8.0)	3347 (26.4)	2293 (21.7)	198 (8.0)	2826 (22.3)	1923 (18.2)	149 (6.0)	2826 (22.3)	1923 (18.2)	149 (6.0)
Total	3699	2548	222	9 798 173.90	6 680 965.20	684 510.90	2900	1955	155	7 858 582.70	5 160 374.10	457 084.20
Mean (SD)	1.1 (0.3)	1.1 (0.4)	1.1 (0.5)	2927.40 (5241.40)	2913.60 (3746.40)	3457.10 (5299.00)	1 (0.2)	1 (0.1)	1 (0.2)	2780.80 (5301.00)	2683.50 (3028.40)	3067.70 (3362.00)
Median (range)	1 (1-4)	1 (1-10)	1 (1-6)	2241.30 (7.80-232 545.80)	2331.70 (6.10-92 658.80)	2309.70 (196.70-58 791.00)	1 (1-2)	1 (1-4)	1 (1-2)	2241.20 (7.80-232 545.80)	2308.20 (23.20-85 302.90)	2270.50 (196.70-31 393.80)
IQR	0	0	0	1436.00	1348.70	1910.00	0	0	0	1332.00	1180.40	1491.40
25th %ile	1.0	1.0	1.0	1584.60	1706.20	1637.20	1.0	1.0	1.0	1588.40	1765.10	1692.90
75th %ile	1.0	1.0	1.0	3020.90	3054.80	3547.50	1.0	1.0	1.0	2920.40	2945.50	3184.40
**ED HCRU and cost**												
N (%)	2312 (18.2)	1833 (17.3)	255 (10.3)	2312 (18.2)	1833 (17.3)	255 (10.3)	1052 (8.3)	854 (8.1)	126 (5.1)	1052 (82.9)	854 (80.8)	126 (50.9)
Total	3699	3003	408	726 342.20	686 855.30	93 521.70	1279	1047	151	299 260.90	322 676.20	49 704.70
Mean (SD)	1.6 (1)	1.6 (1.2)	1.6 (1.2)	314.20 (277.70)	374.70 (343.80)	366.80 (327.70)	1.2 (0.5)	1.2 (0.6)	1.2 (0.5)	284.50 (207.00)	377.80 (266.20)	394.50 (293.50)
Median (range)	1 (1-11)	1 (1-15)	1 (1-11)	229.50 (0.40-3074.60)	285.70 (0.30-3255.00)	293.60 (1.80-2238.20)	1 (1-5)	1 (1-8)	1 (1-3)	250.10 (0.40-1601.70)	340.90 (1.90-2807.10)	362.60 (11.40-1944.70)
IQR	1	1	1	290.80	355.90	381.70	0	0	0	239.10	297.20	351.70
25th %ile	1.0	1.0	1.0	128.70	142.00	128.70	1.0	1.0	1.0	142.60	188.50	180.90
75th %ile	2.0	2.0	2.0	419.50	497.90	510.40	1.0	1.0	1.0	381.70	485.70	532.60
**Outpatient HCRU and cost**												
N (%)	12 065 (95.1)	10 067 (95.2)	2427 (98.1)	12 065 (95.1)	10 067 (95.2)	2427 (98.1)	9879 (77.9)	8386 (79.3)	2244 (90.7)	9879 (77.9)	8386 (79.3)	2244 (90.7)
Total	68 345	53 430	11 579	5 685 795.80	4 250 295.40	985 096.30	19 854	16 324	4273	1 872 400.40	1 475 849.90	394 832.80
Mean (SD)	5.7 (3.9)	5.3 (4)	4.8 (3.4)	471.30 (584.30)	422.20 (632.80)	405.90 (860.20)	2 (1.2)	1.9 (1)	1.9 (0.9)	189.50 (352.80)	176.00 (163.00)	176.00 (138.50)
Median (range)	5 (1-47)	4 (1-84)	4 (1-29)	339.30 (1.00-12 580.80)	299.00 (0.20-32 051.00)	283.70 (3.60-33 273.20)	2 (1-14)	2 (1-13)	2 (1-8)	140.50 (0.40-12 209.60)	150.40 (0.80-4787.50)	161.40 (1.70-2430.20)
IQR	5	5	4	410.00	353.40	297.00	1	1	1	144.10	138.70	128.30
25th %ile	3.0	2.0	2.0	177.10	163.80	173.30	1.0	1.0	1.0	74.80	85.40	95.30
75th %ile	8.0	7.0	6.0	587.00	517.10	470.30	2.0	2.0	2.0	218.90	224.00	223.60

Cohort 1, <2 years of age, n = 12 683; Cohort 2, 2 to <6 years of age, n = 10 573; Cohort 3, 6 to <18 years, n = 2473. Patients could have been present at >1 encounter; hence, the patient counts were not mutually exclusive. Patients/claims with a claim amount of zero were excluded from the analysis. Conversion factor: 1 Arab Emirates Dirham = US $0.272. Gross cost = Insurance paid + Patient share.

Abbreviations: ED, emergency department; HCRU, healthcare resource utilization; IQR, interquartile range; RSV, respiratory syncytial virus; SD, standard deviation; USD: US dollar.

**HCRU and associated cost based on activity type:** For all-cause HCRU based on activity type, in Cohort 1, medications accounted for the largest number of total claims (39 249 claims), followed by services (34 999 claims) and CPT (25 462 claims) (**Table 4**). The total all-cause HCRU cost in Cohort 1 was highest for services (US $5 397 535.70 [median, US $158.90]), followed by CPT (US $3 874 284.80 [median, US $141.20]) and DRG (US $3 557 865.00 [median, US $2167.70]). A similar pattern was reported for disease-specific total claims (**Table 4**). Disease-specific HCRU cost in Cohort 1 was highest for services (US $3 139 805.20 [median, US $72.30]), followed by DRG (US $2 949 196.30 [median, US $2163.20]) and procedures (US $2 357 477.80 [median, US $88.30]). All-cause and disease-specific HCRU and associated costs for different activity types for Cohorts 2 and 3 are summarized in **Table 4**.

**Table 4.** HCRU and Cost in Patients With RSV During 3-Month Post-index Period Based on Activity Type

	**All-cause Claims**			**All-cause Cost (USD)**			**Disease-Specific Claims**			**Disease-Specific Costs**		
	**Cohort 1**	**Cohort 2**	**Cohort 3**	**Cohort 1**	**Cohort 2**	**Cohort 3**	**Cohort 1**	**Cohort 2**	**Cohort 3**	**Cohort 1**	**Cohort 2**	**Cohort 3**
**Procedure HCRU and cost**												
N (%)	11 172 (88.1)	9292 (87.9)	2224 (89.9)	11 172 (88.1)	9292 87.9)	2224 (89.9)	9666 (76.2)	8022 (75.9)	1923 (77.8)	9666 (76.2)	8022 (75.9)	1923 (77.8)
Total	25 462	20 135	4343	3 874 284.80	3 110 771.00	553 718.90	11 825	9348	2157	2 357 477.80	1 771 398.60	305 397.30
Mean (SD)	2.3 (1.6)	2.2 (1.8)	2 (1.4)	346.80 (1106.70)	334.80 (756.70)	249.00 (493.10)	1.2 (0.6)	1.2 (0.5)	1.1 (0.4)	243.90 (1091.50)	220.80 (581.80)	158.80 (203.70)
Median (range)	2 (1-43)	2 (1-76)	1 (1-16)	141.20 (2.20-94990.50)	157.50 (3.30-31679.80)	145.90 (4.00-12502.80)	1 (1-14)	1 (1-6)	1 (1-5)	88.30 (2.20-94990.50)	103.30 (3.30-30855.70)	107.70 (4.00-3353.40)
IQR	2.0	2.0	1.0	278.80	251.20	167.70	0.0	0.0	0.0	152.60	133.80	97.50
25th %ile	1.0	1.0	1.0	67.10	84.00	88.20	1.0	1.0	1.0	45.30	64.20	74.90
75th %ile	3.0	3.0	2.0	345.90	335.20	255.90	1.0	1.0	1.0	197.90	198.00	172.40
**Medication HCRU and cost**												
N (%)	11 633 (91.8)	9587 (90.7)	2299 (92.9)	11 633 (91.8)	9587 (90.7)	2299 (92.9)	8844 (69.7)	7344 (69.5)	1902 (76.9)	8844 (69.7)	7344 (69.5)	1902 (76.9)
Total	39249	30 090	6-160	1 954 252.50	1 094 726.10	261 747.20	12 135	9554	2326	778 811.80	410 291.40	90 082.70
Mean (SD)	3.4 (2.3)	3.1 (2.3)	2.7 (1.9)	168.00 (494.90)	114.20 (192.50)	113.90 (635.90)	1.4 (0.7)	1.3 (0.7)	1.2 (0.5)	88.10 (501.6)	55.90 (133.1)	47.40 (162.2)
Median (range)	3 (1-43)	3 (1-25)	2 (1-18)	93.00 (0.10-36012.00)	69.80 (0.20-7365.20)	59.00 (1.20-29332.50)	1 (1-12)	1 (1-9)	1 (1-6)	32.10 (0.10-36012.00)	25.40 (0.00-5485.40)	22.60 (0.10-6576.40)
IQR	3.0	3.0	2.0	156.00	110.80	95.10	1.0	0.0	0.0	56.70	48.40	33.70
25th %ile	2.0	1.0	1.0	38.80	30.60	27.00	1.0	1.0	1.0	15.20	14.10	14.50
75th %ile	5.0	4.0	3.0	194.80	141.40	122.10	2.0	1.0	1.0	71.90	62.50	48.20
**Consumable HCRU and cost**												
N (%)	1111 (8.8)	604 (5.7)	100 (4.0)	1111 (8.8)	604 (5.7)	100 (4.0)	726 (5.7)	312 (2.9)	50 (2.0)	726 (5.7)	312 (2.9)	50 (2.0)
Total	1334	713	124	123 843.40	56 544.70	20 853.50	754	326	53	81 394.80	21 876.40	5583.60
Mean (SD)	1.2 (0.6)	1.2 (0.6)	1.2 (0.9)	111.50 (330.20)	93.60 (210.10)	208.50 (966.80)	1 (0.2)	1 (0.2)	1.1 (0.2)	112.10 (248.70)	70.10 (132.00)	111.70 (480.90)
Median (range)	1 (1-6)	1 (1-9)	1 (1-8)	38.80 (0.00-8483.10)	34.70 (0.10-2954.30)	25.20 (0.20-8920.40)	1 (1-3)	1 (1-3)	1 (1-2)	47.10 (0.00-3818.50)	25.90 (0.1-1281.2)	18.10 (0.2-3419.5)
IQR	0.0	0.0	0.0	102.50	86.20	96.70	0.0	0.0	0.0	106.10	64.10	40.00
25th %ile	1.0	1.0	1.0	9.80	12.30	12.20	1.0	1.0	1.0	11.10	9.90	14.40
75th %ile	1.0	1.0	1.0	112.30	98.50	108.90	1.0	1.0	1.0	117.30	74.00	54.50
**DRG HCRU and cost**												
N (%)	1360 (10.7)	1272 (12.0)	109 (4.4)	1360 (10.7)	1,272 (12.0)	109 (4.4)	1204 (9.5)	1112 0.5)	87 (3.5)	1204 (9.5)	1112 (10.5)	87 (3.5)
Total	1482	1424	125	3 557 865.00	3 605 535.70	385 714.50	1232	1126	90	2 949 196.30	2 781 010.70	254 539.30
25th %ile	1.0	1.0	1.0	1754.10	1811.40	1902.70	1.0	1.0	1.0	1754.10	1847.00	1916.40
75th %ile	1.0	1.0	1.0	2779.90	2989.10	3379.00	1.0	1.0	1.0	2662.90	2903.80	3327.70
**Services HCRU and cost**												
N (%)	11 769 (92.8)	9613 (90.9)	2240 (90.6)	11 769 (92.8)	9613 (90.9)	2240 (90.6)	9733 (76.7)	7849 (74.2)	1859 (75.2)	9733 (76.7)	7849 (74.2)	1859 (75.2)
Total	34 999	25 017	4849	5 397 535.70	3 002 159.30	401 320.40	11 214	8422	1912	3 139 805.20	1 597 098.40	191 257.80
Mean (SD)	3 (1.9)	2.6 (1.8)	2.2 (1.5)	458.60 (1254.20)	312.30 (957.60)	179.20 (589.20)	1.2 (0.4)	1.1 (0.3)	1 (0.2)	322.60 (1166.30)	203.50 (942.90)	102.90 (535.60)
Median (range)	3 (1-18)	2 (1-19)	2 (1-14)	158.90 (0.70-62308.30)	110.30 (2.70-60497.30)	74.90 (0.20-18628.20)	1 (1-5)	1 (1-4)	1 (1-3)	72.30 (0.50-62308.30)	42.90 (2.70-60240.00)	34.60 (0.20-18248.30)
IQR	2.0	2.0	2.0	307.60	193.10	110.10	0.0	0.0	0.0	93.60	69.60	30.60
25th %ile	2.0	1.0	1.0	73.50	57.20	36.90	1.0	1.0	1.0	34.30	32.70	32.00
75th %ile	4.0	3.0	3.0	381.10	250.30	147.00	1.0	1.0	1.0	127.90	102.30	62.60

Cohort 1, <2 years of age, n = 12 683; Cohort 2, 2 to <6 years of age, n = 10 573; Cohort 3, 6 to <18 years, n = 2473. Patients could have been present at more than one encounter; hence, the patient counts were not mutually exclusive. Patients/claims with a claim amount of zero were excluded from the analysis. Conversion factor: 1 AED = 0.272 USD. Net cost reported is paid by the payers only. Services included consultation (inpatient, emergency department, outpatient, dietitian, physiotherapist, radiology, teleconsultation), daycare services, others (speech therapy, monitoring, physical examinations, laboratory assessments, etc.).

Abbreviations: DRG, Drugs and Diagnosis-Related Group; HCRU, healthcare resource utilization; IQR, interquartile range; RSV, respiratory syncytial virus; USD, US dollar.

**ICU cost:** The ICU cost includes costs incurred for ICU, NICU, and PICU care during the 3-month post-index period. Of the total study population, 376 patients recorded ICU admissions, with the highest representation from Cohort 1 (289 patients). The highest median gross cost was observed for Cohort 2 (US $3510.46 [$905.72-$74 520.10]). However, the total cost was highest for Cohort 1 (US $1 569 330.09). The services included for the determination of ICU costs are outlined in **Table S6**.

## DISCUSSION

RSV infection is a highly prevalent respiratory condition that contributes significantly to the overall disease burden among young children globally. The current retrospective analysis of e-claims data assessed the demographics, treatment patterns, seasonality, comorbidities of interest, risk factors, and direct healthcare costs of RSV infection among individuals aged under 18 years in Dubai, UAE. Although studies in different countries have been conducted to assess the incidence, seasonality, treatment patterns, and economic burden of RSV infection among young children, data from Dubai, UAE, on the clinical and economic burden of RSV infection among young children and adolescents are limited.

In the current study, within the pediatric range of patients <18 years evaluated in the claims data, 49% of patients <2 years and 41% <6 years had an RSV infection, highlighting the burden of RSV disease in young children. Of note, in young children, RSV and influenza are common respiratory illnesses associated with substantial disease burdens. Previous research shows that RSV infections lead to a greater healthcare, economic, and societal burden relative to influenza in young children under 5 years. In young children under 2, retrospective studies have shown that RSV infection is associated with higher rates of ED admissions, hospitalizations, complex hospital courses, respiratory support, and caregiver resource use compared with influenza infection.^19,20^ Furthermore, RSV and influenza are each associated with substantial economic burdens. A cost study from South Africa, which included children under 5 years of age with RSV-associated illness, reported a mean annual cost of US $137 million, of which 76% was healthcare incurred, 6% out-of-pocket expenses, and 13% attributable to indirect expenses.^21^ In another study from South Africa including children under 5 years with influenza-associated illness, mean annual cost was US $270 million, of which 41% were government-incurred costs, 15% was out-of-pocket expenses, and 44% was indirect costs.^22^

The vaccine effectiveness (VE) and immunization coverage for RSV and influenza illnesses in young children differs. The effectiveness of influenza vaccine is well established from public campaigns, while maternal RSV vaccination VE has been established in neonates and young infants, based on clinical trial leading to recent approval (beginning in 2023). Findings from a pooled analysis (n = 24 148; age ≤18 years) of 9 influenza seasons (2011-2020) in the United States showed a VE of 46%, for influenza vaccine.^23^ Immunization rates for influenza was reported as 63.2% in the United States in 2023, while the maternal immunization vaccination coverage for RSV is premature due to the recent launch of these vaccines.^24^

A global epidemiological study from 15 countries, which included 112 seasons of RSV surveillance data between 2000 and 2020, confirmed the high prevalence (55%) of RSV in infants 1 year or younger.^25^ A retrospective analysis of RSV-related hospital admissions in France was conducted in children under 5 years of age. The study findings showed that infants under 1 year of age accounted for nearly 69% of the total RSV infection cases compared with older pediatric age groups.^17^

The current study noted an increase in the number of RSV cases each year from 2014 to 2022, with an average increase of 55% across this period, except for a dip in the number of cases in 2020 by 64% (the overall number of RSV cases reported in 2019 was 4862 and 1753 in 2020). The sudden dip in RSV cases in 2020 could be due to implementation of widespread nonpharmaceutical interventions such as lockdown, travel restrictions, masking, and social distancing, to prevent spread of SARS-CoV-2 virus. The current study subsequently observed a 164% surge in cases in 2021. Several studies noted a similar phenomenon worldwide.^26–28^ It is believed that the dip in RSV cases in 2020 and the sudden surge thereafter was likely due to the introduction, and subsequent relaxation, of nonpharmaceutical public health interventions.

The study outcomes were consistent with the findings from previous retrospective studies.^14,29^

Seasonal variability is another important parameter affecting the prevalence of RSV infection. Notably, RSV infections are more common during the colder seasons, as the virus is reported to have a lower survival rate at high temperatures.^6^ Low temperatures and high precipitation rates play a vital role in promoting viral prevalence. Studies to determine the RSV seasonality in children in the United States or the MENA region observed higher prevalence rates from October to February, corresponding to the winter months.^6,30^ Likewise, in the current study, the percentage of annual RSV cases from January 1, 2014, to December 31, 2022, began to increase across the 3 age cohorts starting in September (8.0%-13.0%) and peaked in October (13.0%-29.0%), November (11.0%-21.0%), and December (9.0%-17.0%).

The most frequent clinical diagnosis observed in the study participants was respiratory disorders, particularly URTIs and LRTIs. In another hospital-based surveillance, fewer respiratory tract complications, particularly bronchiolitis (57.4%) and pneumonia (30.8%), were reported among patients who were RSV positive.^31^

Although infrequent in this study, the primary risk factors in another study for RSV infection among patients under 2 years included congenital disorders and immunodeficiency disorders, neonatal complications, prematurity, and low birth weight.^31^ Key risk factors for RSV episodes, as per the literature, include prematurity, bronchopulmonary dysplasia, congenital heart defects, immune deficiencies, and neuromuscular disorders.^9–11^

The mainstay of RSV treatment is supportive care and symptomatic management, primarily using analgesics, bronchodilators, leukotriene inhibitors, antibiotics, replenishing fluids, and supplemental oxygen.^16,32,33^ Our study also reported similar treatment patterns, with the highest prescription rates for analgesics. Palivizumab, approved in the UAE for immunoprophylaxis of RSV, had a low prescription rate in our study.

The analysis also evaluated the average length of hospital stay, which was found to be around 4 days across all 3 cohorts, in line with previous literature. A retrospective analysis of nationwide hospitalization data conducted in Germany among patients with RSV aged up to 2 years reported the mean length of hospital stay to be 4.5 days.^34^ Similarly, a cross-sectional study conducted in RSV-infected infants, using surveillance registry data from 39 hospitals in the United States, reported a median length of hospitalization of 5 days.^35^

In our study, RSV-associated hospitalization was highest among neonates and young infants less than 2 months old. A retrospective analysis conducted in Italy using a hospital discharge registry also confirmed that the highest RSV infection hospitalization rate (49.6%) was in infants less than 2 months old.^36^ Likewise, in an active surveillance study of RSV-associated hospitalizations in Canada, the highest hospitalization burden was reported for patients aged 2 months or less (37.8%).^37^ According to estimates from a modeling study from the Kingdom of Saudi Arabia, RSV infections resulted in 17 179 to 19 607 hospitalizations of infants, including 2932 to 3675 admissions to the PICU, of which 172 to 525 cases required mechanical ventilation. The total hospitalization cost amounted to SAR 280 million to SAR 340 million (US $75 million–$91 million). Furthermore, the Saudi study estimated that RSV resulted in 57 654 to 191 115 ED visits and 219 083 to 219 970 primary care visits, accounting for 35% of the total economic burden due to RSV.^38^

In the current study, total all-cause and disease-specific costs were highest for Cohort 1. During the 3-month follow-up period, in Cohort 1, the total overall disease-specific cost of RSV, US $10.03 million (median, US $192.20), accounted for approximately 61.9% of the total all-cause cost (US $16.21 million). Major contributors to the high disease-specific cost in Cohort 1 were the total cost of inpatient visits (US $7.85 million) and procedures (US $2.35 million). These factors contributed to 60.0% to 80.0% of their respective total all-cause costs in the 3 months following an RSV diagnosis. Also, the total ICU cost for Cohort 1 (US $1.56 million) was about 2.5 times higher than that for Cohorts 2 (US $529 905) and 3 (US $77 947).

In a population-based retrospective matched cohort study in Ontario, Canada (2006-2016), the total cost for hospitalization among patients with RSV aged ≤2 years over a 10-year period was approximately US $134 million.^39^ A systematic review and meta-analysis reported that the estimated global cost for RSV–acute lower respiratory infection inpatient and outpatient management from a healthcare payer perspective was €4.82 billion (95% confidence interval, 3.47-7.93) among children aged up to 5 years, with approximately 55% of global costs being attributable to hospitalization.^40^ Furthermore, these 2 studies highlighted the greater healthcare cost burden among children with associated comorbidities.

The current study and published literature report a substantial direct cost burden among children with RSV infection. Emerging preventive strategies, such as vaccines and monoclonal antibodies, will play a vital role in reducing the associated clinical burden; however, key evidence gaps exist concerning the economic value of RSV prevention interventions in the region, including the UAE. Additional research on the latest disease burden and cost-effective analysis studies is key to informing payers and healthcare practitioners about adoptive decisions for these new preventive interventions.^33,41^

### Limitations

Despite its findings, the study had certain limitations. The sample size was limited to the privately insured population in Dubai (UAE), which primarily includes expatriates (88.5% of the total UAE population) and a smaller portion of the local Emirati population. However, despite the large expatriate population of expatriates in Dubai, we found that the results will be generalizable. It was not possible to establish sex-based or nationality-based standardization of the data for these demographic outcomes. Additionally, the DRWD records only the year of birth; hence, the patients’ ages were estimated from the birth year and the year of the initial diagnosis. However, we compared our findings among similar age cohorts globally and found them comparable, buttressing the validity of the study. Certain details, such as specific clinical and disease-related parameters (eg, the duration or severity of the illness) and patient mortality figures, were not accessible. In addition, the recorded diagnosis date for RSV infection might not reflect the actual date of diagnosis. For instance, a patient may not have been covered by their insurance plan when they were first diagnosed with RSV. Therefore, the date on which the patient makes the first claim for RSV (for hospital inpatient/outpatient/ED visit, medication, services, consumables, or procedures) during the study period, is considered as the date of diagnosis of RSV for the patient. The claims data documented only conditions, visits, and treatments that occurred while the patients were enrolled in the plan. It is possible that a full medical history, including previous conditions or procedures, was not captured for analysis in this study. Furthermore, the study evaluated only direct healthcare costs; it did not account for indirect costs, such as lost work productivity or quality of life, when assessing the economic impact of RSV infection. The HCRU and cost data are only suggestive since the study did not include any out-of-pocket expenses the patients might have incurred. Additionally, DRWD does not provide a direct linkage between the diagnosis of RSV and the medications, procedures, or consumables used. As a result, the cost reported includes the management of comorbidities treated concurrently with RSV during the visit. Moreover, the patient’s share of expenses for managing RSV is not detailed with regards to costs due to medications, services, supplies, consumables, and procedures; therefore, these data were not included in the HCRU cost assessment for RSV. Also, it was not possible to determine whether the length of the hospital stay was solely due to RSV or other comorbid conditions. The study examined only the treatment patterns, comorbidities, hospitalization, and HCRU/costs for the initial episode of RSV during the index period without considering subsequent reinfections. Furthermore, the current study assessed the treatment pattern, comorbidities, hospital stay, and HCRU/cost of RSV only for the first episode during the index period, and no further assessments were considered at the time of reinfection. This decision was based on the consideration that the above assessments would not materially impact the overall data on HCRU and cost burden among these patients, as RSV is an acute illness that lasts for about 2 to 4 weeks on average.

## CONCLUSION

The current study using the Dubai e-claims database highlights that more than 90% of the RSV burden across Dubai can be attributed to patients under 18 years of age, with half (49%) of the pediatric burden contributed by children aged under 2 years. Furthermore, more than 90% of patients under 2 years of age had an associated respiratory clinical diagnosis, primarily including URTIs, LRTIs, or “other respiratory disorders.” Across the pediatric age range, however, less than 2% of patients in the youngest age group had identified risk factors commonly associated with RSV infection. Between 2014 and 2022, the annual proportion of RSV cases increased by 55% on average. The management of RSV was supportive; as for prophylaxis, less than 1.0% of neonates received palivizumab, a monoclonal antibody, approved against RSV infection in young children with high risk for RSV disease. Disease-specific RSV costs in patients under 2 years of age—largely attributable to inpatient visits, ICU costs, and the costs associated with procedures and services—accounted for 95% of the all-cause cost. Among infants in Cohort 1, 48% of RSV-associated hospitalizations were observed in patients below 3 months of age.

These findings will inform healthcare stakeholders on future policy measures planning for newly available preventive strategies in RSV that could reduce the clinical and economic burden among young children with RSV. Assessing the impact of RSV-related costs can help policymakers to plan and evaluate the cost-benefit of implementing newer methods, such as vaccines, for RSV.

## Supplementary Material

Online Supplementary Material
