# Supplementary material for: Retrospective Analysis of RSV Infection in Pediatric Patients: Epidemiology, Comorbidities, Treatment, and Costs in Dubai (2014-2023)
Source: J Health Econ Outcomes Res. 2024 Nov 5;11(2):133–44. doi: 10.36469/001c.123889 (PMC11580195; doi:10.36469/001c.123889)
Supplement: Online Supplementary Material [file jheor_2024_11_2_123889_252213.pdf]

JHEOR

Journal of Health Economics and Outcomes Research

Online Supplementary Material

Retrospective Analysis of Pediatric Respiratory Syncytial Virus Infections: Epidemiology, Comorbidities, Treatment, and Costs in Dubai (2014-2023). *JHEOR*. 2023;11(2):??-??. doi:10.36469/jheor.2024.123889

Table S1: Codes Selected for RSV Diagnosis

Table S2: Other Respiratory Diseases

Table S3: Length of Hospital Stay

Table S4: Length of Hospital Stay Among Patients in Cohort 1 (≤12 months old) with RSV During 3-Month Post-index (Follow-up) Period

Table S5: Overall HCRU During 3-Month Post-index Period

Table S6: ICU Cost in 3 Months Post-index Period

Figure S1: Distribution of RSV-Associated Hospitalization Among Infants

This supplementary material has been provided by the authors to give readers additional information about their work.

This is an open-access article distributed under the terms of the Creative Commons Attribution 4.0 International License (CCBY-4.0). View this license's legal deed at http://creativecommons.org/licenses/by/4.0 and legal code at http://creativecommons.org/licenses/by/4.0/legalcode for more information.

Table S1. Codes Selected for RSV Diagnosis

ICD Code	Diagnosis
B97.4	Respiratory syncytial virus as the cause of diseases classified elsewhere
J12.1	Respiratory syncytial virus pneumonia
J20.5	Acute bronchitis due to respiratory syncytial virus
J21.0	Acute bronchiolitis due to respiratory syncytial virus

Abbreviation: ICD, *International Classification of Diseases*; RSV, respiratory syncytial virus.

Table S2. Other Respiratory Diseases

ICD Code	Diagnosis
R05	Cough
R06.2	Wheezing
R06.02	Shortness of breath
J84.114	Acute interstitial pneumonitis
J98.01	Acute bronchospasm
R06.03	Acute respiratory distress
J84.115	Respiratory bronchiolitis interstitial lung disease
R06.89	Other abnormalities of breathing
P28.89	Other specified respiratory conditions of newborn
R06.09	Other forms of dyspnea
R09.82	Postnasal drip
J96.90	Respiratory failure, unspecified, unspecified whether with hypoxia or hypercapnia
J96.02	Acute respiratory failure with hypercapnia
J98.8	Other specified respiratory disorders
J69.0	Pneumonitis due to inhalation of food and vomit
J96.01	Acute respiratory failure with hypoxia
R06.1	Stridor
J98.19	Other pulmonary collapse
P27.1	Bronchopulmonary dysplasia origin in the perinatal period
J96.91	Respiratory failure, unspecified with hypoxia
J96.92	Respiratory failure, unspecified with hypercapnia
J98.9	Respiratory disorder, unspecified
J99	Respiratory disorders in diseases classified elsewhere
P28.5	Respiratory failure of newborn
R06.5	Mouth breathing
I26.99	Other pulmonary embolism without acute cor pulmonale
J69.8	Pneumonitis due to inhalation of other solids and liquids
J81.0	Acute pulmonary edema
J81.1	Chronic pulmonary edema
J84.10	Pulmonary fibrosis, unspecified
J84.112	Idiopathic pulmonary fibrosis
J84.113	Idiopathic non-specific interstitial pneumonitis
J96.00	Acute respiratory failure, unspecified whether with hypoxia or hypercapnia
P28.2	Cyanotic attacks of newborn
T17.590A	Other foreign object in bronchus causing asphyxiation, initial encounter

Table S3. Length of Hospital Stay

Parameter	Cohort 1	Cohort 2	Cohort 3
No. (%) of patients	2826 (22.3)	1923 (18.9)	149 (6.0)
No. (%) of claims	2900	1955	155
Length of hospital stay, days			
Mean (SD)	4.1 (3.4)	3.8 (4.2)	4.2 (4.2)
Median (range)	4.0 (1.0 to 95.0)	3.0 (1.0-130.0)	3.0 (1.0-38.0)
IQR	2.0	1.0	1.0
25th %ile	3.0	3.0	3.0
75th %ile	5.0	4.0	4.0

Cohort 1, <2 years of age, n = 12 683; Cohort 2, 2 to <6 years of age, n = 10 573; Cohort 3, 6 to <18 years, n = 2473.
Abbreviations: IQR, interquartile range; SD, standard deviation.

Table S4. Length of Hospital Stay Among Patients in Cohort 1 (≤12 mo old) with RSV During 3-Month Post-index (Follow-up) Period

Age of Patients (mo)[a]	No. (%) of Patients (N = 2826)[b]	No. (%) of Claims (N = 2900)[b]	Length of Stay			
			Mean (SD)	Median (Range)	IQR	25th and 75th %ile
1	581 (20.6)	596 (20.6)	4.8 (5.9)	4.0 (1.0-95.0)	2.0	3.0, 5.0
2	522 (18.5)	537 (18.5)	4.2 (2.7)	4.0 (1.0-36.0)	2.0	3.0, 5.0
3	252 (8.9)	260 (8.9)	4.0 (1.8)	4.0 (1.0-12.0)	2.0	3.0, 5.0
4	235 (8.3)	237 (8.2)	3.8 (1.6)	3.0 (1.0-15.0)	2.0	3.0, 5.0
5	186 (6.6)	192 (6.6)	4.1 (3.7)	3.0 (1.0-36.0)	2.0	3.0, 5.0
6	142 (5.0)	148 (5.1)	3.9 (2.4)	3.0 (2.0-20.0)	1.0	3.0, 4.0
7	165 (5.8)	172 (5.9)	4.1 (2.4)	3.0 (1.0-21.0)	2.0	3.0, 5.0
8	138 (4.9)	141 (4.9)	3.9 (2.1)	3.0 (1.0-15.0)	2.0	2.0, 4.0
9	104 (3.7)	107 (3.7)	4.0 (2.0)	3.0 (2.0-12.0)	1.0	3.0, 4.0
10	106 (3.8)	109 (3.8)	4.0 (1.9)	4.0 (1.0-11.0)	2.0	3.0, 5.0
11	88 (3.1)	89 (3.1)	3.7 (1.5)	3.0 (2.0-9.0)	1.0	3.0, 4.0
12	87 (3.1)	88 (3.0)	3.5 (2.0)	3.0 (2.0-19.0)	2.0	2.0, 4.0

Study period: Jan. 1 2014–Sept. 30, 2023.
Index period: Jan. 1 2014–June 30, 2023.
[a]Age of patients in Cohort 1 was calculated based on the assumption that a patient was 30 days old when the first RSV claim was made in the DRWD.
[b]Patients or claims with length of stay data available in the RSV claims.
Abbreviations: DRWD, Dubai Real-world Database, IQR, interquartile range; RSV, respiratory syncytial virus; SD, standard deviation.

Table S5. Overall HCRU During 3-Month Post-index Period

	Overall All-cause Claims			Overall All-cause Cost (USD)			Overall Disease-Specific Claims			Overall Disease-Specific Costs (USD)		
	**Cohort 1**	**Cohort 2**	**Cohort 3**	**Cohort 1**	**Cohort 2**	**Cohort 3**	**Cohort 1**	**Cohort 2**	**Cohort 3**	**Cohort 1**	**Cohort 2**	**Cohort 3**
No. (%) of patients counted	12 681 (99.9)	10 572 (99.9)	2473 (100.0)	12 681 (99.9)	10 572 (99.9)	2473 (100.0)	12 677 (99.9)	10 570 (99.9)	2 473 (100.0)	12 677 (99.9)	10 570 (99.9)	2 473 (100.0)
Total	75 743	58 981	12 209	16 210 311.90	11 618 115.90	1 763 128.90	24 033	19 326	4 579	10 030 244.10	6 958 900.20	901 622.80
Mean (SD)	6 (4.1)	5.6 (4.2)	4.9 (3.6)	1278.30 (3078.50)	1099 (2399.20)	713 (2375.80)	1.9 (1.2)	1.8 (1.0)	1.9 (0.9)	791.20 (2755.50)	658.40 (1628.00)	364.60 (1083.20)
Median (range)	5 (1-49)	4 (1-86)	4 (1-30)	524.30(0.40-232545.80)	432.90 (0.80-124709.80)	317.00 (3.60-77020.50)	2 (1-14)	2 (1-13)	2 (1-8)	192.20 (0.40-232545.80)	192.00 (0.80-85441.80)	171.70 (1.70-31393.80)
IQR	5	6	4	1281.20	915.00	378.20	1	1	1	417.00	301.90	142.90
25th %ile	3	2	2	252.30	217.70	192.50	1	1	1	106.20	117.60	111.90
75th %ile	8	8	6	1533.50	1132.70	570.70	2	2	2	523.20	419.50	254.80

Cohort 1, <2 years of age, n = 12 683; Cohort 2, 2 to <6 years of age, n = 10 573; Cohort 3, 6 to <18 years, n = 2473.
Note: The overall HCRU and associated costs present cumulative data of encounter type and activity type assessments. Patients/claims with a claim amount of zero were excluded from the analysis.
Conversion factor: 1 Arab Emirates Dirham = 0.272 USD
Abbreviations: HCRU, healthcare resource utilization; IQR, interquartile range; SD, standard deviation; USD, US dollar.

Table S6. ICU Cost in 3 Months Post-index Period

**ICU Gross Cost[a] (USD)**	**Cohort 1**	**Cohort 2**	**Cohort 3**
No. (%) of patients	289 (2.3)	78 (0.7)	9 (0.4)
Total cost	1 569 330	529 905	77 947
Mean cost (SD)	5430.21 (8055.43)	6793.65 (10,797.29)	8660.74 (9649.58)
Median cost (range)	2838.09 (691.82-78,929.99)	3510.46 (905.72-74,520.10)	3184.36 (1339.12-28,982.61)

Cohort 1, <2 years of age, n = 12 683; Cohort 2, 2 to <6 years of age, n = 10 573; Cohort 3, 6 to <18 years, n = 2473.
Note: Patients could have been present at >1 encounter; hence, the patient counts were not mutually exclusive. Patients/claims with a claim amount of zero were excluded from the analysis.
[a]ICU cost includes cost incurred for ICU, NICU, and PICU care. Gross Cost = Insurance Paid + Patient Share.
Abbreviations: ICU, intensive care unit; NICU, neonatal intensive care unit; PICU, pediatric intensive care unit; SD, standard deviation; USD, United States dollar.

Figure S1. Distribution of RSV-Associated Hospitalization Among Infants

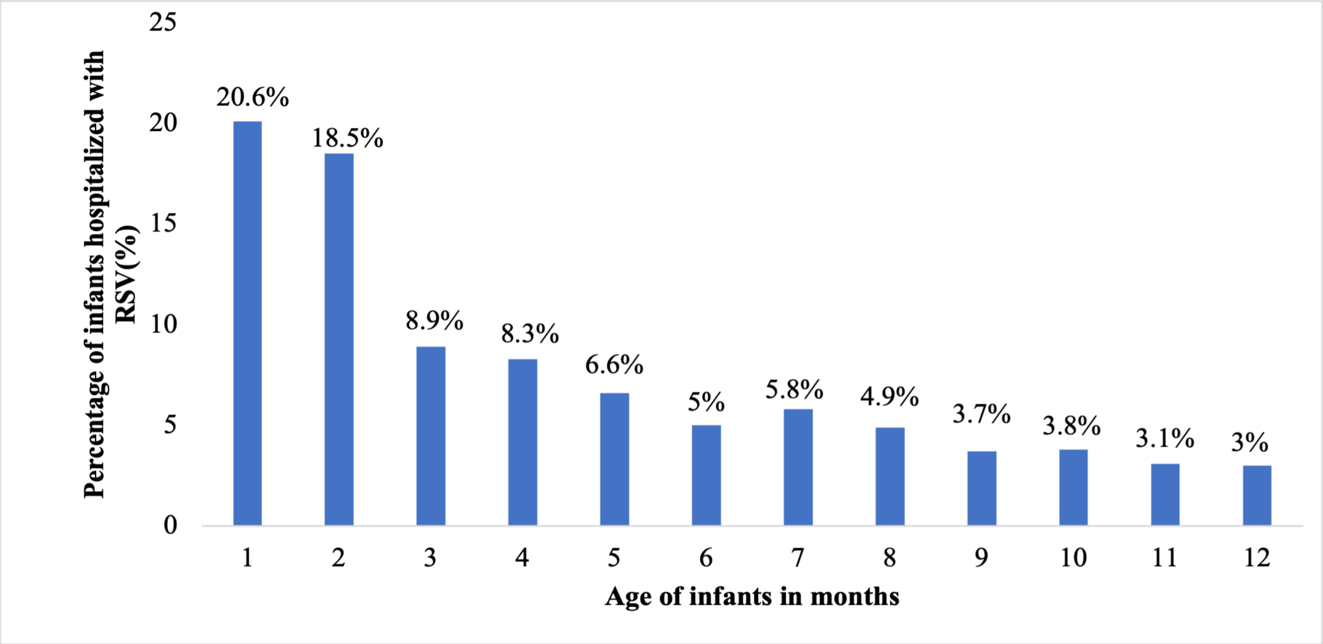

Abbreviation: RSV, respiratory syncytial virus.
